# Supplementary material for: Deletion of wfs1 Impairs Oligodendrocyte Precursor Cells Dorsal Distribution and Myelination Through the wfs1-hmgcs1 Axis in Zebrafish
Source: Biology (Basel). 2026 Aug 16;15(16):1402. doi: 10.3390/biology15161402 (PMC13510151; doi:10.3390/biology15161402)
Supplement: Supplementary file 1 [file biology-15-01402-s001.zip › File S1-Transcriptome sequencing data and differentially expressed genes (DEGs) in wfs1-- zebrafish at 5 dpf/2.KEGG/wfs1KOvsWT/ALL/src/dre00515.html]

dre00515


- dre:569769

- Down regulated genes

569769(-6.48720906679522)

- dre:100093713

- Down regulated genes

100093713(-3.03530509299907)

- dre:569769

- Down regulated genes

569769(-6.48720906679522)

- dre:569769

- Down regulated genes

569769(-6.48720906679522)

- dre:100093713

- Down regulated genes

100093713(-3.03530509299907)

- dre:569769

- Down regulated genes

569769(-6.48720906679522)

- dre:569769

- Down regulated genes

569769(-6.48720906679522)

- dre:569769

- Down regulated genes

569769(-6.48720906679522)

- dre:569769

- Down regulated genes

569769(-6.48720906679522)

Close
